# Case Report: Identification of a Novel *GNAS* Mutation and 1p/22q Co-Deletion in a Patient With Multiple Recurrent Meningiomas Sensitive to Sunitinib

**DOI:** 10.3389/fonc.2021.737523

**Published:** 2021-10-15

**Authors:** Weiping Hong, Changguo Shan, Minting Ye, Yanying Yang, Hui Wang, Furong Du, Xing Zhang, Chao Song, Linbo Cai

**Affiliations:** ^1^Department of Oncology, Guangdong sanjiu Brain Hospital, Guangzhou, China; ^2^The State Key Laboratory of Translational Medicine and Innovative Drug Development, Jiangsu Simcere Diagnostics Co., Ltd., Nanjing, China; ^3^Department of Medicine, Nanjing Simcere Medical Laboratory Science Co., Ltd., Nanjing, China

**Keywords:** refractory meningioma, *GNAS* mutation, 1p/22q co-deletion, sunitinib, next-generation sequencing

## Abstract

**Background:**

Although surgical resection can cure the majority of meningiomas, there are still approximately 20% of patients suffering from an aggressive course with recurrence or progression. In this study, we reported a novel *GNAS* mutation and 1p/22q co-deletion responding to sunitinib in a patient with multiple recurrent meningiomas.

**Case Presentation:**

A 53-year-old woman with meningioma was hospitalized due to postoperative tumor progression for 3 weeks. WHO grade I meningioma was pathologically diagnosed after the first three surgeries, but the second recurrence occurred approximately 3 years following the third surgery. Next-generation sequencing was performed on the first two recurrent samples. *GNAS* mutations and 1p/22q co-deletion were both identified, and amplification at 17q and chromosome 19 was also found in the second recurrent sample, based on which WHO grade II/III meningioma was diagnosed. The lesion in the left cerebellopontine angle area enlarged after use of radiotherapy combined with temozolomide chemotherapy for 2 months. When sunitinib was added, the residual lesions began to lessen and continuously reduced.

**Conclusion:**

This typical case suggested that timely molecular diagnosis for refractory meningiomas contributed to guiding the molecular classification and clinicians to make more reasonable individualized therapeutic regimens, consequently benefiting the patients. This case report also highlighted the potential role of sunitinib in the treatment of refractory meningiomas.

## Introduction

Meningiomas primarily arising from meningothelial arachnoid cells are the most common intracranial tumors at present, with an estimated annual prevalence of 8.83 cases per 0.1 million people in the Central Brain Tumor Registry of the United States (1). Although surgical resection can cure the majority of meningiomas, there are still approximately 20% of patients suffering from an aggressive course with recurrence or progression, leading to increased morbidity and mortality (2).

With advances in the molecular characterization of meningiomas, the genetic aberrations that may be potential treatment targets and those representing an elevated risk of tumor recurrence have been identified. Apart from *NF2* alterations in sporadic meningiomas, various oncogenic mutations in *KLF4*, *SMO*, *TRAF7*, *PIK3CA*, *AKT1* and *POLR2A* are also found to be clinically actionable genetic events in meningiomas (3–5). Additionally, mutations in *AKT1*, *SMO* and *TERT* promoter may indicate an increased likelihood of tumor recurrence (6, 7). Although surgery and/or irradiation are the major treatment modality for aggressive and/or recurrent meningiomas, these new genetic findings provide potential targets for drug treatment. Here, we shared a novel *GNAS* mutation and 1p/22q deletion responding to sunitinib in a patient with multiple recurrent meningiomas.

## Case Presentation

**Figure 1:**
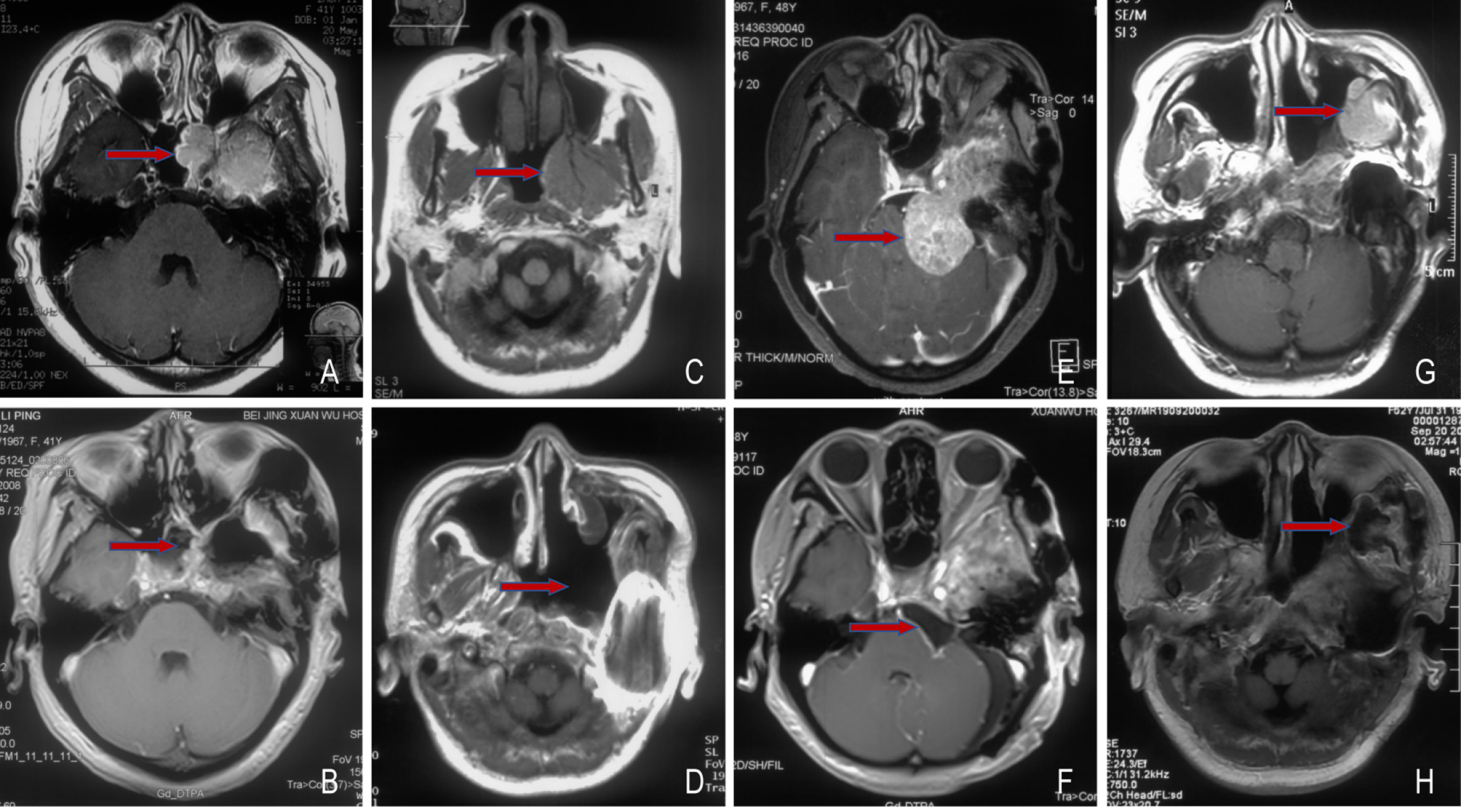
Changes of the pre- and post-operative meningioma. Before **(A)** and after **(B)** the first surgery, 2008-06; Before **(C)** and after **(D)** the second surgery, 2009-01; Before **(E)** and after **(F)** the third surgery, 2016-05; Before **(G)** and after **(H)** the fourth surgery, 2019-09. The red arrow points to the tumor.

A 53-year-old woman with meningioma was admitted to our hospital due to postoperative tumor progression for 3 weeks. In May 2008, she had decreased hearing in the left ear, abnormal secretion in the left nasal cavity and a water-flow sensation in the laryngopharyngeal wall. Magnetic resonance imaging (MRI) showed a space-occupying lesion between the left cavernous sinus and the left pterygopalatine fossa (**Figure 1A**). In June 2008, she underwent meningioma resection in orbitozygomatic-infratemporal approach (**Figure 1B**). Immunohistochemical results showed EMA (+), CK (-), Vimentin (+) and Ki-67 (5%). The pathological diagnosis was WHO grade I endothelial meningioma. Postoperatively, the patient still had decreased hearing, facial paralysis, fixed eyeballs and facial numbness in the left side, and discharged from hospital after recovery.

In January 2009, MRI revealed a residual tumor (**Figure 1C**), thus the tumor resection in the left nasopharyngeal wall was performed (**Figure 1D**). Immunohistochemical results indicated EMA (+) and Vimentin (+). WHO grade I transitional meningioma was diagnosed according to pathological results. Postoperatively, the left facial paralysis, fixed eyeballs and facial numbness of the patient were almost the same as before, and gradually improved after rehabilitation exercises.

In January 2016, the patient started to have headache and dizziness accompanied by left blurred vision. One month later, MRI showed a recurrent tumor involving cavernous sinus in the left cerebellopontine angle area (**Figure 1E**). In May 2016, partial excision of meningioma in the left skull base was conducted (**Figure 1F**). Immunohistochemical results showed EMA (+), Vimentin (+), GFAP (-), PR (+) and Ki-67 (5%). WHO grade I transitional meningioma was pathologically diagnosed. Postoperatively, headache and dizziness relieved, without obviously aggravated left blurred vision. The patient refused to receive adjuvant radiotherapy due to personal reasons. Further consultations indicated stable disease.

In July 2019, the patient had mild swelling in the left canthus, soft in texture. One month later, MRI indicated a recurrent tumor with distant diffusion (**Figure 1G**). In September 2019, cranio-orbital resection of space-occupying lesions based on the right fronto-orbital-zygomatic initial incision was performed under general anesthesia (**Figure 1H**). Through immunohistochemistry, it was found S-100 (-), Vimentin (+), EMA (+), ER (-), PR (+), SSTR2a (+), E-Cadherin (+), INI-1 (+), Ki-67 (5%-15%), EGFR (+++) and GFAP (-). The pathological results showed WHO grade I-II transitional meningioma. The patient did not receive further treatment after surgery.

**Figure 2:**
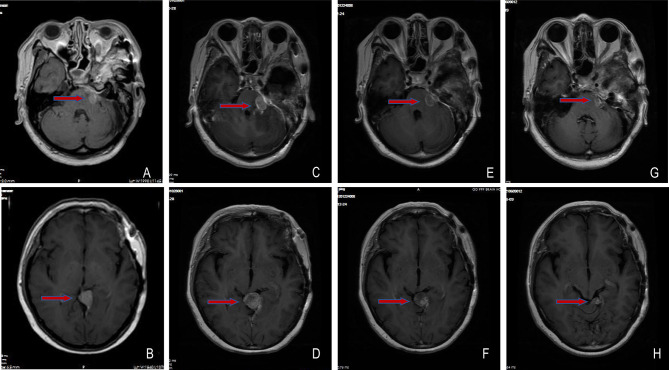
Changes of meningioma at different shooting angles of MRI. **(A, B)** Meningioma recurred on 2020-07-23; **(C, D)** The lesions in the left cerebellopontine angle area enlarged on 2020-10-28; **(E, F)** The residual lesions lessened on 2020-12-24; **(G, H)** The residual lesions continuously reduced on 2021-06-09. The red arrow points to the tumor.

**Figure 3:**
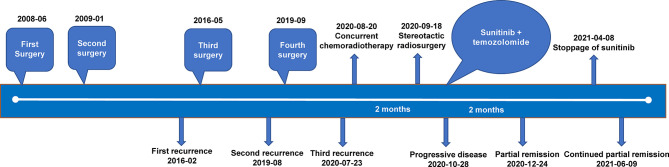
The treatment timeline of the patient with multiple recurrent meningiomas.

On July 23, 2020, tumor progression was observed. The patient came to our hospital for further diagnosis and treatment. Her symptoms, such as left decreased hearing, left facial numbness, right deviated corners of the mouth and limited left eye movement in vertical direction, were the same as before. MRI showed an abnormal enhancement ratio in the relative left brainstem and an enhanced lesion under the cerebellar tentorium, suggesting tumor recurrence and distant diffusion (**Figures 2A, B****)**. Next-generation sequencing (NGS) was performed on the first two recurrent samples, and somatic inactivating mutations in *GNAS* gene and 1p/22q co-deletion were both identified. Additionally, amplification at 17q and chromosome 19 was also found in the second recurrent sample. These findings suggested high-grade meningioma (WHO grade II/III). Concurrent chemoradiotherapy (prescribed dose: 40 Gy/20f, temozolomide: 75 mg/m^2^) was applied on August 20, 2020. Stereotactic radiosurgery (SRS) boost was used for lesions under the tentorium of cerebellum (8 Gy) and in the left cerebellopontine angle area (5 Gy) on September 18, 2020. Two months after chemoradiotherapy, the lesion in the left cerebellopontine angle area enlarged (**Figures 2C, D****)**, which might be associated with response to radiotherapy in a short term. Then, sunitinib (50 mg/d) was orally administrated in combination with temozolomide chemotherapy, the residual lesions lessened after use of 2 months (**Figures 2E, F****)**. Sunitinib was totally applied for four treatment courses, in which 6 weeks were as one treatment course. To date of June 9, 2021, MRI showed continued partial remission of residual lesions (**Figures 2G, H****)**. The patient was in good condition, and symptoms like facial paralysis and fixed eyeballs improved. The treatment timeline of the patient is depicted in **Figure 3**.

## Discussion

Although most meningiomas are benign and have good outcomes after surgical resection, a small subset of them is likely to recur, which requires repeated surgeries, radiation and medical treatments (8). Recently, emerging evidence has revealed the association of recurrent mutations with a particular clinicopathological phenotype in meningiomas, and the updated WHO classification of brain tumors has also incorporated some important molecular findings (2, 9). The case in our study was pathologically diagnosed as WHO grade I meningioma with an extremely low 5-year recurrence rate after the first three surgeries, but recurrence occurred approximately 3 years following the third surgery. Through genetic testing, *GNAS* mutations and 1p/22q co-deletion were both identified in the first two recurrent samples.

GNAS, a crucial signal transduction protein, can activate adenylate cyclase during the signal transduction of G-protein-coupled receptors, leading to increased cyclic adenosine monophosphate levels (10). *GNAS* is mutationally activated in various cancer types, such as growth hormone-secreting pituitary tumors, pancreatic cancer and colorectal cancer, while has inactivating mutations in pseudohypoparathyroidism type 1a (11, 12). In our study, this inactivating *GNAS* mutation was first found in meningioma. However, whether this mutation affects meningioma progression needs more studies to confirm.

Structural and numerical chromosome variations are accompanied by more aggressive features of meningiomas, most significantly 1p deletion, and the deletion of the distal part of 1p is correlated with meningioma progression (2, 13). Moreover, aberrations of several other chromosomes, such as 22q, 6q, 14q and 17q, are reported to involve in meningioma development and progression (14). Although WHO grade I meningioma was pathologically diagnosed in our study after the third surgery, 1p/22q co-deletion was detected at molecular levels, suggesting a probability of tumor recurrence. The patient should be followed up closely. Compared with the first recurrence, the second recurrence was accompanied by more chromosomal variations including amplification at 17q and chromosome 19, which highlighted more complicated changes of molecular genetics in the second recurrent sample. These genetic findings provided the evidence for WHO grade II/III meningioma.

The patient experienced 3 recurrences in total after 4 surgeries, which might be associated with absence of positive adjuvant therapies including SRS that are known to have high rates of tumor control when used to small tumor remnants (15). Based on the results of molecular diagnosis, the regimen of radiotherapy plus temozolomide chemotherapy + anti-angiogenic targeted therapy was made. However, the patient rejected to receive anti-angiogenic targeted therapy until an enlarged lesion appeared, and the residual lesions significantly lessened after use of sunitinib. As an orally administered small tyrosine kinase inhibitor targeting vascular endothelial growth factor receptor (VEGFR), KIT and platelet-derived growth factor receptor, sunitinib has been demonstrated active in patients with recurrent malignant meningioma in a phase II trial where the 6-month progression-free survival (PFS) rate in the cohort of anaplastic and atypical meningiomas was 42% and the expression of VEGFR2 in the tumor tissue was associated with favorable PFS (16). Moreover, sunitinib showed similar efficacy and safety in the systemic management of refractory meningiomas compared with octreotide/everolimus (17).

In conclusion, this typical case suggested that timely molecular diagnosis for refractory meningiomas contributed to guiding the molecular classification and clinicians to make more reasonable individualized therapeutic regimens, consequently benefiting the patients. This case report also highlighted the potential role of sunitinib in the treatment of refractory meningiomas. Whether the effect of sunitinib on refractory meningiomas will be translated into improved disease progression including improved PFS and overall survival is yet to be determined.

## Data Availability Statement

The original contributions presented in the study are included in the article/supplementary materials. Further inquiries can be directed to the corresponding authors.

## Ethics Statement

Written informed consent was obtained from the individual(s) for the publication of any potentially identifiable images or data included in this article.

## Author Contributions

WH performed the data acquisition and wrote the manuscript. CGS, MY, YY, and HW performed the radiological images analysis. FD and XZ collected the data of genetic testing. CS and LC reviewed the manuscript. All authors contributed to the study and approved the submitted version.

## Conflict of Interest

Authors FD, XZ and CS were employed by Jiangsu Simcere Diagnostics Co., Ltd. and Nanjing Simcere Medical Laboratory Science Co., Ltd.

The remaining authors declare that the research was conducted in the absence of any commercial or financial relationships that could be construed as a potential conflict of interest.

## Publisher’s Note

All claims expressed in this article are solely those of the authors and do not necessarily represent those of their affiliated organizations, or those of the publisher, the editors and the reviewers. Any product that may be evaluated in this article, or claim that may be made by its manufacturer, is not guaranteed or endorsed by the publisher.
